# FlyDetector—Automated Monitoring Platform for the Visual–Motor Coordination of Honeybees in a Dynamic Obstacle Scene Using Digital Paradigm

**DOI:** 10.3390/s23167073

**Published:** 2023-08-10

**Authors:** Yuanyuan Huang, Guyue Lu, Wei Zhao, Xinyao Zhang, Jiawen Jiang, Qiang Xing

**Affiliations:** 1 School of Mechanical Engineering, Nantong University, Nantong 226019, China; 2 Shanghai Aerospace System Engineering Institute, Shanghai 201108, China

**Keywords:** automatic, honeybee behavior, bio-vision, optic flow, image acquisition

## Abstract

Vision plays a crucial role in the ability of compound-eyed insects to perceive the characteristics of their surroundings. Compound-eyed insects (such as the honeybee) can change the optical flow input of the visual system by autonomously controlling their behavior, and this is referred to as visual–motor coordination (VMC). To analyze an insect’s VMC mechanism in dynamic scenes, we developed a platform for studying insects that actively shape the optic flow of visual stimuli by adapting their flight behavior. Image-processing technology was applied to detect the posture and direction of insects’ movement, and automatic control technology provided dynamic scene stimulation and automatic acquisition of perceptual insect behavior. In addition, a virtual mapping technique was used to reconstruct the visual cues of insects for VMC analysis in a dynamic obstacle scene. A simulation experiment at different target speeds of 1–12 m/s was performed to verify the applicability and accuracy of the platform. Our findings showed that the maximum detection speed was 8 m/s, and triggers were 95% accurate. The outdoor experiments showed that flight speed in the longitudinal axis of honeybees was more stable when facing dynamic barriers than static barriers after analyzing the change in geometric optic flow. Finally, several experiments showed that the platform can automatically and efficiently monitor honeybees’ perception behavior, and can be applied to study most insects and their VMC.

## 1. Introduction

When performing complex tasks for long periods, it is challenging for micro-UAVs to accomplish adaptive sensing tasks in unknown environments owing to the lack of abundant onboard resources [1]. In contrast to compound-eyed insects, they exhibit highly sophisticated flight behaviors such as collision avoidance [2], hunting [3], and spouse localization [4], despite their relatively small brains and limited processing capacities [5]. Optic flow is the primary and critical source of spatial information used by insects to recognize obstacles and exhibit adapt their flight behavior [6]. When confronted with obstacles, insects can actively control their magnitude of motion to change the optical flow input to the visual system and ultimately simplify visual processing [7,8]. The visual perception VMC mechanism contained in the recognized obstacles of insects matched well with the required perception scheme of the micro-UAV. Therefore, flight robots are expected to achieve efficient detection of visual perception [9], inspired by VMC, when mimicking insects by actively controlling the magnitude of motion to change the input of the visual system to simplify visual processing.

Previous studies have shown that insect behaviors can be triggered by artificial external stimuli [10], and based on this, a large number of systems have been extensively used to explore many VMCs of insects [11,12,13,14]. Thus, flight channels equipped with high-speed cameras are widely used. For example, in the experiment of Ravi et al. [15], the flight channel and camera acquisition platform were used to analyze the behavior sequence images of a single bumblebee crossing a barrier wall gap, and parameters such as the centroid position, body length, and heading of the bumblebee during gap crossing were extracted through image processing. In addition, the vector velocity changes during the flight of the bumblebee were integrated to study the mechanism of identifying gaps and evaluating their passage during flight. For example, Bertrand et al. [16] studied the interactions of visual memory, collision avoidance, and path integration of bumblebees using intra-channel heterochromatism to determine whether bumblebees use motor and visual memories to solve navigational conflict situations. Utilizing high-speed schlieren photography and particle-tracking-velocimetry, the wake flow of tethered houseflies was investigated [17], and Convolutional Neural Networks (CNNs) were also used to predict bee behaviors [18]. In the above experiments, high-speed cameras were used at a uniform high frequency, which can easily cause data collection (large amounts of data) and complex problems. Although many artificial stimuli acquisition devices have been developed to study the perceptual mechanisms of flight visual impairments in different compound-eyed insects, most rely on complex, labor-intensive operations, which are time-consuming and inefficient.

Although these devices have been developed to study the VMC behavior of flying insects, most are complicated manual operations. In particular, existing methods rely heavily on unintuitive theoretical calculations of optic flow. Therefore, in the process of analyzing the vision of compound-eyed insects, it is often assumed that the intersection points between the instantaneous light on the insect retina and the flight tunnel walls are used to calculate the instantaneous intersection point with the spatial position and direction of the bumblebee retina and the total geometric light flow of the small eye. Although this simplified optical flow estimation method is similar to the insect optical flow estimation, it depends on the theoretical calculation of the optical flow, and there may be a deviation in the optical flow variation information in the actual process. Previous studies have shown that insects depend heavily on active visual stimuli to guide their flight, such as collision avoidance [12], guided landing, and speed control [19]. Analysis of the trajectory data of insects crossing gaps showed that *Apis cerana* preferred a wider aperture and a brightness strategy was used to guide the flight of insects through the gaps. Moreover, as the gap visibility and contrast of optical flow decreased, insects spent more time identifying gaps. The geometry and pass ability of the gap can be evaluated using repeated lateral maneuvers [13]. In particular, active lateral maneuvers are more extensive and faster for narrower gaps. Autonomously controlled migration can enhance boundary or spatial recognition [20] to ensure that insects pass smoothly through narrow gaps. Thus, flying insects can shape the corresponding photosensitive inputs through specific movements or changes in perspective when performing tasks. Additionally, few devices are available for studying the behavior of insects in the presence of dynamic obstacles, leading to a lack of behavioral data in dynamic environments and making studies on the VMC of insects in dynamic environments difficult.

This study was conducted to design an unsupervised device to monitor the VMC of insects that overcomes the limitations of previous studies and provides a virtual mapping system to visually investigate the mechanism of insects [21], which can reconstruct the optical flow information of the global field of view from the main insect perspective using mapping. The application potential of this system was determined by studying the VMC behavior of honeybees (*A. cerana*) in the presence of a dynamic obstacle, and the results show that the optical flow image reconstructed by the virtual mapping system is consistent with the theoretical calculation, which proves that the system can be used to study the VMC mechanism of insects.

## 2. Materials and Methods

**Figure 1:**
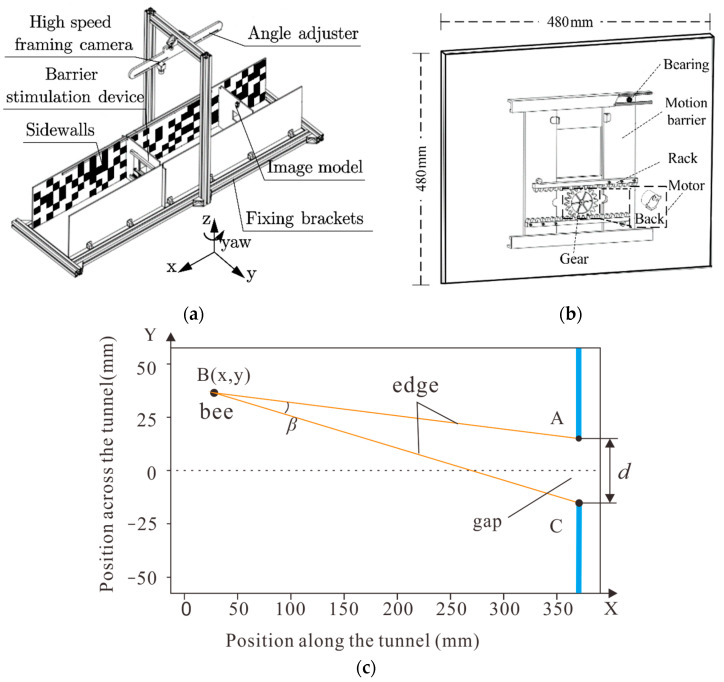
(**a**) The structure of automatic monitoring platform. (**b**) Mechanical assembly diagram of the barrier stimulation device, where *d* means the gap’s actual size in horizontal direction. (**c**) The position of the descriptions, where “B” represents the position of the bee while “A “and “C” respectively represent the two boundaries of the gap.

The automated monitoring platform designed to investigate the VMC behavior of insects, as shown in Figure 1a, comprised four systems: A visual disturbance stimulation system, an automatic-trigger image-recording system, an insect attitude and obstacle position analysis system, and a virtual mapping system.

### 2.1. Visual Disturbance Stimulation System

This system was comprised of a barrier stimulation device and a flight tunnel (1.56 × 0.28 × 0.28 m). The tunnel side walls were lined with a red and white random pattern following a 1/*f* frequency distribution, similar to those described in [15]. The barrier stimulation device was the core of the visual stimulation system and included a stepper motor, driver, and gear rack, as shown in Figure 1b. The stepper motor with gears was fixed at the back of the barrier, and the gap size in the horizontal direction (*d*) changed with the gear meshing and rack when the motor rotated. The direction and speed of the motor can be adjusted by adjusting the pulse frequency and number. Based on the meshing principle, Equation (1), the linear velocity of the gap is alternately set to move left or right at a constant speed:

(1) $$v_{c}=\left(2\pi n/60\right)\times r_{c}=\frac{2\pi f_{p}\times r_{c}}{i}$$

where $v_{c}$ is the linear velocity of the gap, n is the rotational speed of the stepper motor, $f_{p}$ is the pulse frequency used to control the stepper motor, $r_{c}$ is the radius of the gear-indexing circle, and the reduction ratio i of the stepper motor is 64.

Then the theoretical distance from the obstacle can be calculated using the formula $D=\frac{d}{tan\left(\delta /2\right)}$, where *D* represents the obstacle distance, *d* is the gap width, and δ is the honeybee’s ommatidia visual angle. It is suggested that the visual angle of honeybees’ ommatidia is approximately 5° [22]. Therefore, the length of the tunnel must be greater than twice the obstacle distance.

### 2.2. Automatic Trigger Image Recording System

**Figure 2:**
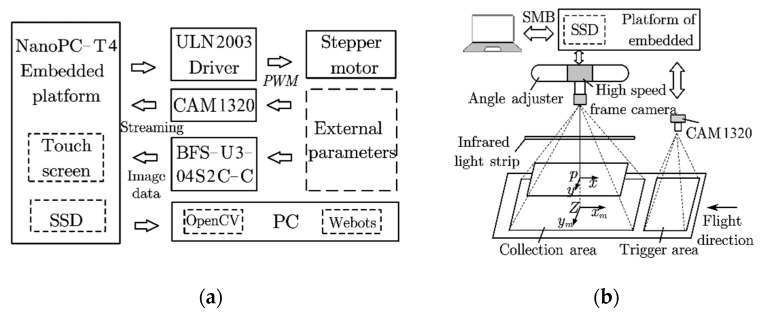
(**a**) Structure diagram of the control system. (**b**) Schematic diagram of the capture process.

This system included an embedded platform (NanoPC-T4, Guangzhou Friendly Electronic Technology Co., Ltd., Guangzhou, China) with a wide-angle detection camera (CAM1320), a high-speed framing camera (BFS-U3-04S2C-C, Teledyne FLIR, Wilsonville, OR, USA), a solid-state disk (Pro 512G, Gloway, Shenzhen, China), and an infrared lamp (24 W, wavelength 940, red mirror) as supplementary lighting (Figure 2). For the embedded platform to trigger a high-speed framing camera, the MOG2 function [23] was adapted to segment the background of the images from the CAM1320. After calculating the segmented image, the ‘ContourArea’ function was used to calculate the area of the insect contour in the images, and the position of the centroid of the insect was calculated using the ‘Moment’ function. The pixel coordinates of the centroid were then stored in an array of length *n*. When the array was full, the increase or decrease in the array was determined using Equation (2).

(2) $$P_{x_{S}}=\frac{\sum_{1}^{n}\left(P_{x_{i}}-P_{x_{i-1}}\right)}{n}$$

where n is the storage queue length, $P_{x_{i}}$ is the x-pixel centroid of the contour in the previous frame, and $P_{x_{i-1}}$ is the x-pixel centroid of the contour in the current frame.

In the case of an array increment, the initialized integer variable, Flag, was incremented. After the insect completely passed through the Cam1320 field of vision, the positive and negative values of Flag were determined to confirm the movement direction of the insect. To ensure the accuracy of contact, the number and size of the ‘ContourArea’ functions were limited.

When the insect’s movement direction matched the settings, the high-speed framing camera was triggered to capture sequential images of the insect crossing the gap. For successful capture, the high-speed framing camera was equipped with a factory automation camera lens (FA0801C, f: 8 mm, shutter speed: 1/1.8′, the field of view: 46.8° × 36°), and the sampling rate was 163 frames per second (fps).

### 2.3. Insect Attitude and Obstacle Position Analysis System

**Figure 3:**
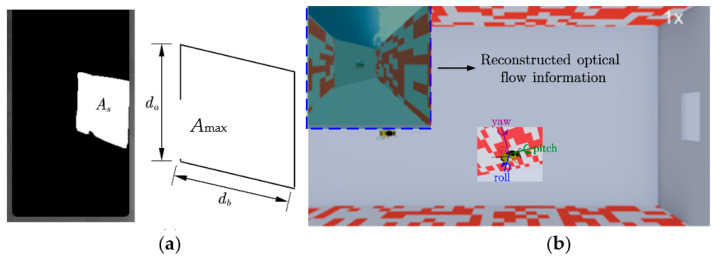
(**a**) Schematic diagram of gap calculation. (**b**) Mapping of visual perceptual motion.

This system consists of a PC with the OpenCV and Server Message Block (SMB) protocol. The sequence images captured using the high-speed framing camera were synchronized with a PC using the SMB. Next, attitude data, including the position and yaw angle of the insects, were analyzed by calculating the sequence images using OpenCV (Script 1); the obstacle’s gap size was calculated, and the results were saved in CSV format. For data analysis, the computer configuration was as follows: Intel (R) Courier (TM) i7-12700 @ 3.50 GHz CPU, 16.00 GB memory. It was difficult to ensure that the pulse rate of the motor was synchronized with the sampling rate of the high-speed camera; thus, the gap conversion method of the motor could not be used to obtain information on the gap, trajectory, and attitude of the insect simultaneously. To ensure the synchronization of the collected information, the VMC behavior of the compound-eyed insects was mapped more accurately. When acquiring insect pose information, OpenCV was used to calculate the ROI region of dynamic obstacles in the sequence image. The specific process is as follows:

First, the location of the dynamic obstacles was selected within the first frame image, and background segmentation was then performed on the images within the selected ROI region. The ‘findContours’ function was used to extract the contour information of the white area, and the ‘contourArea’ function was used to calculate the pixel area size of the contour information. When the door plate was moved, the distance of the segment *d*_b_ in the horizontal direction changed, while in the vertical direction *d*_a_ remained the same. Finally, according to the quadrilateral area, the gap distance of the current frame can be calculated using Equation (3).

(3) $$d_{s}=\frac{A_{s}}{A_{m}{}_{ax}}d_{\max}$$

where $d_{s}$ is the gap’s actual size in the horizontal direction of the current frame, $A_{s}$ is the actual pixel area under the current frame, $A_{max}$ is the maximum pixel area in the image, and $d_{\max}$ is the actual maximum horizontal distance of the gap (Figure 1b and Figure 3a).

### 2.4. The Virtual Mapping System

This system was primarily composed of a virtual platform Webots (open-source robot simulation software developed by Cyberbotics). A virtual scene was created to retain the insects’ real flight environment, where the obstacles changed as in the real environment, and an insect model was built to simulate the motion of an insect in the virtual environment (Script 2). The flight states depend on the attitude data of the actual insects and the mapping effect (Figure 3b).

### 2.5. Operation Process Using the Platform

The use of this platform consisted of three primary operating steps: Setup and adjustment, data processing, and mapping simulations. In the setup and adjustment steps, the positions of the camera and light source were adjusted based on the proper installation of the relevant software. The motor mode was set on an embedded platform to generate visual stimuli for the appropriate motion. In the data processing stage, the visual stimulation system caused the honeybees to adjust their behavior, and the magnitude of the visual motion experienced. Subsequently, the automatic-trigger video-recording system captured the behavior of the honeybees’ VMC and saved the data on a solid-state disk when the honeybees passed through the detection area in the specified direction. Third, the information obtained based on the insect’s attitude and using an obstacle position analysis system was examined. Finally, the honeybees’ visual information collected by the sensors installed in the virtual model was used to assess the VMC mechanisms.

## 3. Experiments

To verify the performance of the platform at different flight speeds and compare the accuracy of manual and automatic acquisition, we used speed simulation software (Script 3) to conduct several experiments at the Intelligent Detection and Biomimetic Technology Research Laboratory of Nantong University on 20 June 2022.

### 3.1. Accuracy and Applicability Experiments of the Platform

**Figure 4:**
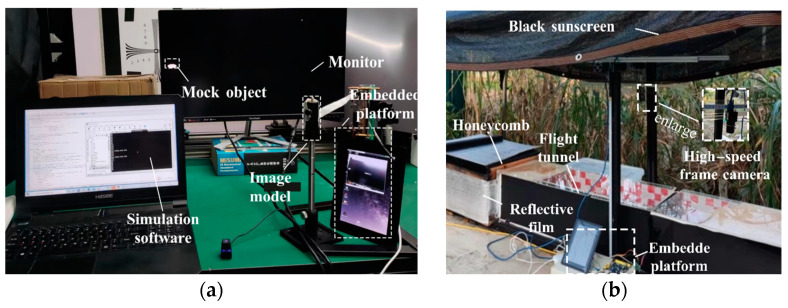
(**a**) Speed adaptability experiment of the trigger module. (**b**) Motion trajectory map collected in the actual scene and virtual environment.

Experiment 1: Speed adaptability of the trigger module. This experiment was designed to test the impact of the platform’s accuracy on different speed targets. First, a monitor (VX2780-2K-Pro, 170 fps, ViewSonic, Brea, CA, USA) was placed 25 cm away from the image model to display moving mock objects, as shown in Figure 4a. Second, the number of collections was set to 20. The movement interval of the mock object was set to 30 s. An automatic-trigger image-recording system was used to detect the movement of the mock object. We tested a speed range of 1–12 m/s three times and counted the data collected at each speed.

**Figure 5:**
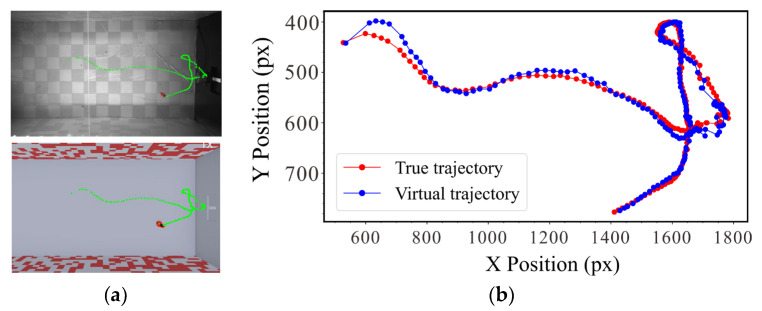
(**a**) Accuracy and efficiency of automatic acquisition. (**b**) Comparison of motion trajectories in real and virtual environments.

Experiment 2: Mapping effect of the platform. Because a single high-speed camera was used to collect the visual perception behavior of the insects, the virtual mapping process was based on the height in the middle of the channel, which resulted in a loss of height information. To verify the error of the system in mapping the actual insect flight behavior, we extracted the trajectories of insect flights from the sequence images, as shown in Movie 1. The actual and virtual environments and the pixel errors between the corresponding trajectory points were compared (Figure 5).

### 3.2. Field Record Experiments of Honeybees

Data collected in the field were used to verify the performance of the platform, and experiments were conducted on honeybees at the Southern Plain Corn Observation Station of Nantong University from 20–27 June 2022. The experiments were conducted using individual honeybees (*A. Cerana* 1793) from a single colony. Primary nectar sources, such as maize, rapeseed, and auxiliary nectar, are sufficient around the observatory, which is conducive to the survival and reproduction of honeybee colonies. The honeycomb was placed on a platform (45 cm above the ground) and covered with a reflective film to simulate the natural habitat of honeybees (Figure 4b). In addition, black sunscreen was installed above the platform to avoid direct sunlight, which led to high temperatures inside the honeycomb.

Experiment 3: Accuracy and efficiency of automatic acquisition. The opening between the hive and the tunnel was blocked with cotton to prevent honeybees from exiting the hive, and the camera was focused to ensure that the image was clear. The honeybees were allowed to adapt to the expected infrared sensation for one week. The automatic collection system was turned on when honey bees (>20) constantly flew through the tunnel. Both sides of the tunnel were used to control the movement of the bees, allowing only one honey bee to enter the tunnel at a time, and the next honey bee was released after data were captured and extracted from the image.

Experiment 4: Dynamic and static experiment. The purpose of this experiment was to determine the VMC of honeybees facing various obstacles. Honeybees were allowed free entry into the flight tunnel to ensure that the platform automatically monitored flight behavior without human interference. The static experiment was conducted as described in Experiment 3. Many worker honeybees (>20) could fly continuously in search of food; thus, the static obstacle was set 30 cm away from the tunnel exit, and the automatic monitoring platform was used to capture sequential images. The capture time was set to 1 h to ensure that the insects encountered the obstacle for the first time; multiple honeybees flying an incomplete flight were considered invalid images. The dynamic obstacle experiments were conducted one week after removing the obstacle to avoid the influence of the static experiment. 

### 3.3. Data Analysis

In the analysis of the VMC of honeybees facing dynamic and static obstacles, 10 sets of flight attitude data were analyzed. Because the data were not from a single honeybee, we performed a paired *t*-test to compare the variation in mean parameters within the same individuals of the population, such as the average contrast of the optic flow generated by the honeybee’s head trajectory. All statistical tests were performed using Python, and statistical significance was set at Student’s *t*-test, *p* < 0.05.

## 4. Discussion

### 4.1. Accuracy and Applicability of the Platform

**Figure 6:**
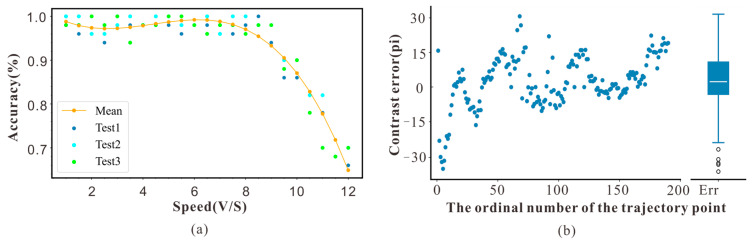
(**a**) Accuracy data of experimental device at different target speeds. (**b**) Error analysis diagram of corresponding pixel coordinates.

The results of Experiment 1 are shown in Figure 6. The results indicated that when the simulated target speed was <8 m/s, the trigger accuracy of the device was as high as 98%, and the detection accuracy was stable. However, the detection accuracy decreases at least quadratically when the speed is >8 m/s. This is because the frame rate of the CAM1320 was 30 fps, whereas the maximum distance provided by the monitor was 620 mm. When the speed was >8 m/s, the number of detection frames was less than two, and the algorithm failure led to a reduction in the trigger accuracy. Therefore, the speed range of the collection trigger system for detecting objects should be <8 m/s, and the typical cruising speed of honeybees flying in an outdoor environment is approximately 7 m/s; thus, the system could be used to detect the movement of honeybees.

The results of Experiment 2 are shown in Figure 6b. We compared the actual trajectory with the mapped trajectory. The horizontal axis represents the trajectory point, and the vertical axis represents the error between the two trajectories. The results showed that the error between the mapped and actual trajectories was within 15 pixels, demonstrating that this system can map the complex flight behavior of insects.

### 4.2. Field Record Experiments of Honeybees

**Figure 7:**
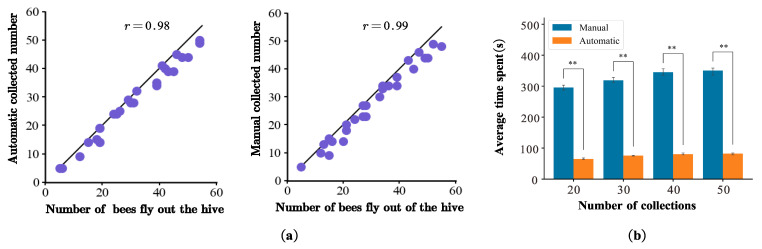
(**a**) Comparison of basic facts of accurate triggering between automatic and manual methods; represents the number of insects passed and the number of actual trigger collection. (**b**) Efficiency comparison of automatic and manual operations. Double asterisks indicate significant differences (Student’s *t*-test, *p* ≤ 0.001).

The results of Experiment 3 obtained through manual and automatic collection in the field were highly consistent (Figure 7a). The manual accuracy rate was 0.99, and the automatic accuracy rate was 0.98. The automatic collection method was similar to the ground truth in terms of the number of collections. Manual image capture is time-consuming in terms of efficiency. At least 6–7 min was required to extract the motion parameters for each honeybee. In contrast, only 1–2 min were required to complete the extraction using the automatic collection system, which resulted in a 2–4-fold increase in efficiency (Student’s *t*-test, *p* < 0.001, Figure 7b). Therefore, the automated acquisition platform performed well without intensive manual operation.

**Figure 8:**
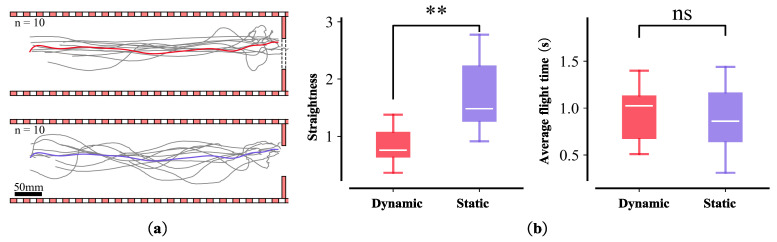
Dynamic and static experiment. (**a**) Flight trajectory under dynamic obstacles (the number of bees is 10); red line shows the fitting of the average flight trajectory ((**a**)-top). Flight trajectories under static obstacles (n = 10); purple line shows the fit of the average flight trajectory ((**a**)-bottom). (**b**) Comparisons of time and straightness of flight between two scenes. Box plots (median, upper, and lower quartiles) showing the dispersion of the mean trajectory of the bees for the first ((**b**)-left) and second ((**b**)-right) average flight time of two objects under dynamic and static obstacles conditions. n.s. (non-significant; *p* > 0.0125); double asterisks indicate significant differences (Student’s *t*-test, ** *p* ≤ 0.0025).

The flight trajectory of the honeybees was relatively stable under dynamic conditions but varied significantly under static obstacles (Figure 8a). We averaged the fitting of the flight trajectories under the two conditions and analyzed the dispersion and duration of each flight trajectory (Figure 8b). The results revealed no significant difference in flight time between the two conditions, whereas the degree of dispersion of the flight trajectories differed significantly.

**Figure 9:**
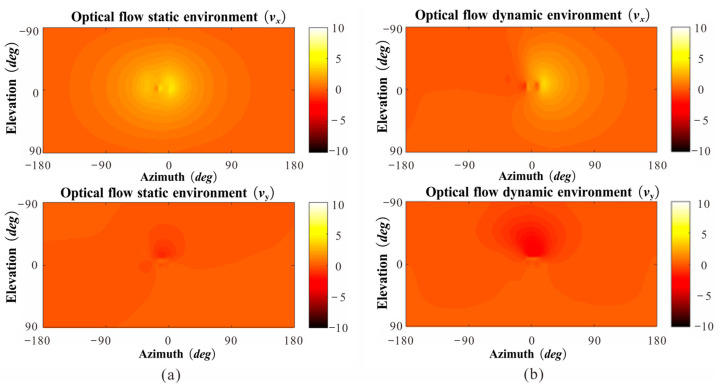
Sample snapshot of the *V*_x_, *V*_y_ geometric optic flow change for a full spherical field of view at a given moment during the flight. (**a**) Separately computed using the time course of the flight trajectory and orientation of the head. (**b**) Separately computed using the image from the virtual mapping system.

We analyzed the instantaneous vision of honeybees using a virtual mapping system and attempted to explain the phenomenon in which insects fly more stably in the presence of dynamic obstacles than in the presence of static obstacles. The results show that the visual instantaneous directional optic flow, $V_{x}$, was more evident than $V_{y}$ when facing a static obstacle (Figure 9a). Thus, we hypothesized that honeybees actively strengthen their horizontal motion amplitude when facing static obstacles, causing the optic flow generated by the edge of the gap to expand significantly on their forehead. However, when facing a dynamic obstacle, the honeybees move far less in the lateral direction than when facing a static obstacle (Figure 9b). The change in direction was consistent under both static and dynamic conditions. Unlike in the static case, the change in optical flow under dynamic situations occurred primarily because of the gap movement. Therefore, honeybees may actively reduce the magnitude of their visual motor when objects move relative to themselves because the optic flow stimuli distinguished the obstacles.

A new analysis system named FlyDetector was developed to automatically record data on insect behavior and was used to map the flight process to evaluate the mechanism of VMC. Several traditional methods, including tethered flight systems [14], insect flight mills [24], and obstacle flight tunnels with high-speed cameras [15], have been used to examine the mechanisms of VMC behavior. However, these methods are time-consuming. Our system overcomes these limitations by automatically monitoring insect flight. The experimental results confirm the usefulness of the proposed system.

Our platform includes a virtual mapping system that uses a virtual environment to map the physical motions of insects. This system can improve the analysis of VMC behavior in insects. Installing various sensing devices on insects is difficult because of their small size, which makes it challenging to directly obtain insect behavioral parameters. Alternatively, virtual insect entities can also be used. In this study, we used a fisheye camera to simulate optic flow changes on insect foreheads and analyzed the collected images of the entire visual field to study the VMC of insects. In addition, we determined three parameters of honeybee behavior, including *x*- and *y*-axis differences and yaw orientation, which have been widely used to describe VMC behavior, such as collision avoidance [25,26] and visual memory behaviors [16,27]. In addition, real-time parameters of the gap were supported, enabling studies on the VMC mechanism in insects facing movement barriers.

Three classical visual parameters were used to analyze the behavior of compound-eyed insects [16,28], in order to verify our hypothesis that the flight stability of compound-eyed insects when facing dynamic scenes was due to them actively reducing the accuracy of the amplitude of the lateral visual motion recognition behavior. These included retinal size γ (γ, Equation (4)), rate of change of retinal size $\dot{\gamma }$ ($\dot{\gamma }$, Equation (5)), and relative retinal expansion velocity RREV (RREV, Equation (6)). Each parameter was evaluated for each instant of the flight segments and compared with the time course of the honeybees.

(4) $$(\begin{array}{l} \alpha =\arctan(\frac{C_{y}-B_{y}}{C_{x}-B_{x}})\\ \beta =\arctan(\frac{A_{y}-B_{y}}{A_{x}-B_{x}})\\ \gamma \left(t\right)=\alpha -\beta \end{array}$$

(5) $$\dot{\gamma }\left(t\right)=\frac{d\gamma }{dt}$$

(6) $$RREV\left(t\right)=\frac{\dot{\gamma }\left(t\right)}{\gamma \left(t\right)}$$

In the equation, B represents the position of the bee, while A and C respectively represent the two boundaries of the gap, as shown in Figure 1c.

**Figure 10:**
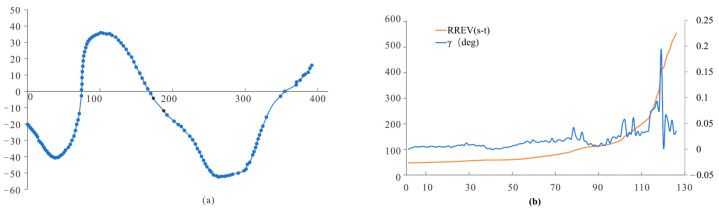
(**a**) Static scene flight trajectories; (**b**) visual parameter analysis.

In the static obstacle scenario, the included angle of the retina of compound-eyed insects tended to increase continuously, and *RREV* (*t*) fluctuated. When *RREV* (*t*) approaches zero, as shown by the green, pink, and red dots (Figure 10), the corresponding flight paths are all located in the early stages of motion deflection. Therefore, the change in the retinal dilation rate *RREV* (*t*) is related to the VMC of compound-eye insects. It can be inferred that when the expansion rate of retinal dilation *RREV* (*t*) of compound-eye insects approaches zero, insects improve their retinal dilation rate *RREV* (*t*) by changing their lateral movement behavior to enhance the recognition of static obstacles. This is consistent with the conclusion that insects recognize obstacles by enhancing lateral flight motion in static scenes.

**Figure 11:**
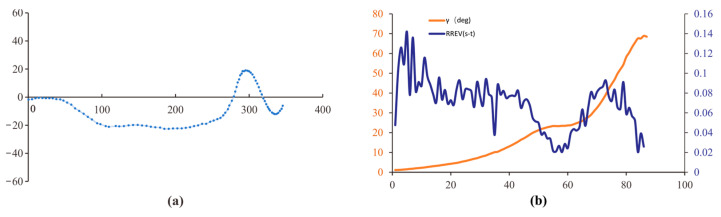
(**a**) Dynamic scene flight trajectories; (**b**) visual parameter analysis.

In dynamic scenes, when the dynamic gap increased, the changes in the retinal size of compound-eyed insects also tended to increase; however, the overall range of changes in *RREV* was much larger than that in static scenes. Similar to the static scenes, when *RREV* (*t*) approaches zero, the green, pink, and red dots (Figure 11) indicate that the corresponding flight paths are also located in the early stage of motion deflection, but their deflection amplitudes are smaller than those in the static scene. Combined with the flight path of the honeybee, when the dynamic gap increases, the increase in interstitial motion stimulates frontal light flow in the head of the honeybee to compensate for the deficiency of frontal light flow stimulation through lateral motion. This also explains why compound-eyed insects fly smoothly in dynamic situations. Comparing the results of the dynamic and static scenes, *RREV* plays an important role in the obstacles faced by compound-eyed insects. When the optical flow stimulation provided by external obstacles cannot meet the recognition conditions of compound-eyed insects, they enhance their recognition of obstacles by strengthening lateral flight movement. When the light flow stimulation provided by external obstacles meets the recognition conditions for compound-eyed insects, the insects weaken this behavior appropriately.

The use of a monitoring platform to study the flight behavior of insects showed that honeybees use lateral scanning behavior to confirm their position and judge the passability of obstacles, which is consistent with previous results [20]; however, this behavior is not apparent when facing dynamic obstacles. Another study [19] suggested that honeybees are susceptible to apparent differences in virtual gap mapping. When an obstacle moves, the optical flow change at the gap edge becomes pronounced [29], which may stimulate the honeybee’s forehead by changing the solid optical flow. When the optic flow stimulus meets the requirements for obstacle recognition, honeybees actively reduce the intensity of lateral scanning. Furthermore, regardless of changes in the insect’s flight position in the tunnel, they always detected gaps with their heads facing light and kept bright areas in their field of view. Therefore, optical navigation strategies are practical under dynamic conditions.

## 5. Conclusions

We developed an automated monitoring device that can provide dynamic obstacle visual stimuli while recording the VMC behavioral data of insects. After mapping the virtual mapping system, the visual movement behavior of the insects was studied from various perspectives. Using this system, we found that insect flight was more stable in the presence of dynamic obstacles than in the presence of static obstacles and inferred that this characteristic is optic flow variation. The virtual mapping detector provides a scalable foundation for studying flight behavior for various purposes and insect species, such as identifying insect movement disorders and visual memory mechanisms of flight movement.

Next, we described the limitations and future extensions of our platform in terms of the following aspects: (1) The applicability of speed, (2) insect clustering, and (3) changing 2D trajectory to 3D. The frame rate determines the sampling rate of the automatically triggered image-recording system, which is typically 60 fps. This is the speed limit for the detected objects. To overcome this problem, the tunnel size can be adjusted. Lecoeur et al. [30] changed the distance and density of obstacles in a tunnel to control the optical flow on both sides to affect the flying speed of a bumblebee. Therefore, the collection accuracy can be improved by changing and narrowing the virtual tunnel mapping to reduce the flight speed of insects.

During field collection, because the bees were in a colony, we collected flight sequence images of multiple honeybees, which led to fewer data for each individual. Long-term data collection can compensate for this limitation. Determining the collective behavior of multiple honeybees crossing a gap is important for studying insect colony behavior. Moreover, the hardware performance of the development platform was limited, such as the number of USB 3.0 interfaces and virtual mapping support, and ultra-high-speed simultaneous acquisition of multiple high-speed cameras could not be supported. Thus, the device can only obtain two-dimensional pose information, resulting in the loss of flight height and spatial altitude behavior parameters. Although height parameters have little influence on the VMC behavior of insects, it is necessary to obtain these spatial parameters for the spatial mapping of virtual mapping systems.

## Data Availability

Not applicable.
